# An Immunological Perspective to Non-syndromic Sensorineural Hearing Loss

**DOI:** 10.3389/fimmu.2019.02848

**Published:** 2019-12-11

**Authors:** K. P. Sindura, Moinak Banerjee

**Affiliations:** Neurobiology and Genetics Division, Rajiv Gandhi Center for Biotechnology, Thiruvananthapuram, India

**Keywords:** Non-Syndromic Hearing Loss, congenital, immune system, pathogen, immunogenetics, inner ear, environmental factors, host response

## Abstract

Conventionally the etiology of congenital Non-Syndromic Hearing Loss has been attributed to mutations in the genes involved in ion homeostasis or the structural compartments of the inner ear. However, this contributes to only a part of the problem, as still the determinants for a large majority of the Non-Syndromic Hearing loss seems to be an enigma. Evidences indicate that pathogens like Rubella, Cytomegalovirus, and many other infections can also result in congenital hearing loss. Additionally, there are variety of factors other than the viral mediators, that can act as stressors to trigger an altered immune response, during the gestational period of the mother. It is also known that non-specific stimulation of the immune system can mimic an infection status. This indicates a strong role for environmental factors toward their contribution to the pathology, possibly by influencing the host immune response. These varieties of known or unknown environmental factors interact with the susceptible variants in immune response genes in defining the threshold for protection or infection in an individual. Considering this background we propose to present this perspective that threshold of the host immune response during the prenatal conditions, in response to environmental stimulus, might be determined by the susceptible variants in immune response genes. This in turn can directly or indirectly influence the genes involved in maintaining the structural components or ion homeostasis, resulting in hearing loss. The threshold of immune response alterations may be heavily dependent on the immunogenetic profile of the mother or the fetus.

## Introduction

Congenital hearing loss is one of the most prevalent sensory deficit in the world affecting one out of 650 newborns (1). Based on broad phenotype the congenital hearing loss is classified into Syndromic hearing loss and Non-Syndromic Hearing loss (NSHL). NSHL accounts for 70% of congenital hearing loss (2) with 75–80% of them showing autosomal recessive mode of inheritance (3). The etiology of the disease is largely attributed to genetics but still a large majority of NSHL cases are devoid of major pathogenic mutations in the major contributor genes. Even the pathology shows high genetic and clinical heterogeneity and thereby increases the complexity in understanding the biological basis of the disease. Evidences suggest that environmental factors like Rubella, Cytomegalovirus (CMV) infections, and Acquired Immunodeficiency syndrome can also direct toward the causation for the disease. The development of inner ear occurs during the first trimester of pregnancy (4). Any intrinsic or extrinsic perturbations at this developmental stage, be it genetic or environmental can result in hearing loss. This paved our interest to delve into the unexplored domain of immunogenetics or environmental impact on the role of immune system in hearing loss.

## Genetic Contribution to Nshl and its Heterogeneity

The genetic spectrum of NSHL has been elaborately studied in different populations of the world and till date 85 causative genes have been discovered (5) (Supplementary Figure 1). Mutations in *GJB2* which encodes for connexin 26, was the first to be identified to have a role in NSHL (6). It is involved in forming gap junctions in inner ear, which is crucial in maintaining the ion homeostasis of the inner ear (7). Although this gene is still the most prominent causative factor for autosomal recessive NSHL (3) but the spectrum of *GJB2* mutation differs in different populations of the world. In Caucasian population *GJB2* mutations are attributed to 50% of NSHL, with c.35delG being the most prevalent (8), while in Asian populations *GJB2* mutations account for only 16%, with c.235delC being the major mutation (9–11). In Ashkenazi Jews population c.167delT is the major causative mutation (12). Apart from these frequent mutations, *GJB2* harbors around 140 mutations responsible for the causation of the disease (davinci.crg.es/deafness). This opens up for a debate as to what contributes to hearing loss pathology in rest of the population. A recent Deafness Variation Database (deafnessvariationdatabase.com), identifies 152 genes implicated in syndromic and non-syndromic deafness and reports that <1% of the variants are pathogenic or likely to be pathogenic in nature (13). This comprehensive database comprises of 876,139 variants and classifies 7,502 (0.85%) as pathogenic, 671 (0.077%) as likely pathogenic, 15,287 (1.74%) as likely benign, 156,970 (17.9%) as benign, and 695,709 (79.4%) as variants of uncertain significance. Among these variants 96% of coding variants are rare and novel and that the pathogenicity is driven by minor allele frequency thresholds, variant effect, and protein domain. Therefore, on one side the ethnic specific variants within the same gene with relatively high penetrance ranging from 16 to 50%, while the remaining part of the story is made up of mutations in other genes with low penetrance possibly acting as a cumulative factor. We would therefore like to argue that this cumulative factor might be mediated by environment or environmentally controlled genetic factor.

## Environmental Perspective in Nshl and their Immunological Trigger

While causative genes do impact hearing loss but the role of environmental factors also cannot be ruled out. CMV, Rubella infections, Congenital Toxoplasmosis, Lymphocytic Choriomeningitis virus, Trepenoma pallidum, and Acquired Immunodeficiency syndrome are known infectious agents that can cause acquired NSHL (3, 14). CMV and Rubella infections during the first trimester increases the predisposition risk of congenital hearing loss. The exact means by which these infection results in hearing loss is not yet completely known. However, few studies have reported alterations in endolymph concentration and direct cochlear damage to be the causation (15). Rubella infection can show direct cytolytic effect on the fetus or induce infection derived immune responses in the mother, fetus, and placenta, which can elevate the proinflammatory state resulting in the causation of disease (16). It has been reported that RV-IgM antibody testing which is determined for Rubella infection can also be induced by non-specific stimulation of the immune system (17). Infact, there could be many other environmental factors that could trigger a similar proinflammatory response but timing and duration of the proinflammatory response resulting in a hearing loss pathology will be determined by the host immunogenetic parameters, and its subsequent direct or indirect interaction with the pre-disposing genes for hearing loss. Immunogenetic parameters have been reported to have differential impact on rubella infection (18). These altered immune response can be critical in determining protection to rubella infection as differential cytokine induction impact the production of Immunoglobulins (19). Therefore, while these viral mediators are known to result in hearing loss, we would like to argue that a similar pro-inflammatory response can be mediated by many other environmental stressors too, and the threshold to withstand these environmental factors might be determined by host immunogenetic parameters.

## Immune System in Ear

Immune system alterations in developing hearing loss was never the prime choice for various investigators as inner ear was believed to be an immune privileged organ until 1979, because of the presence of blood labyrinth barrier. Rask-Anderson and Stahle discovered contact between macrophages and lymphocytes in endolymphatic sac of guinea pigs (20). In the same year, McCabe also described autoimmune inner ear disease (21). Adams in 2002 demonstrated the presence of cytokine receptors in cochlear cells and reported that type I fibrocytes of spiral ligament were the main producer of cytokines in the inner ear (22). These facts clearly indicate that the inner ear is susceptible to immune mechanisms of the host.

## Molecular and Cellular Players for Immune Response Modulation

Considering that there is a strong genetic and environmental component to NSHL, and knowing that human ear is no more an immune privileged organ, makes us to propose that genetics of immune response and its phenotypes should also be considered to understand NSHL. It is known that a wide range of heritable and non-heritable factors can perturb the immune system and a combined understanding of these heritable and non-heritable influences on immunity is necessary to understand the inter-individual variation and its consequences on health and disease. The composition of immune cells varies among different populations. A recent study has reported racial differences in activation of B cell receptor signaling pathway in healthy individuals (23). Another study again on healthy cohorts have shown significant differences in relative frequencies of six principal immune cell populations: B cells, monocytes, natural killer (NK) cells, CD4+ T cells (22–90%), CD8+ T cells, and total T cells (24). Gene expression profiling of T cells and dendritic cells revealed that 22% of the overall variation in gene expression between individuals could be attributed to heritable factors (25) and these heritable factors are also implied in pathogen sensing (26).

Initially the genetic effect of both humoral and cellular immune responses was first attributed to MHC genes (27) and is known to be the most polymorphic region in humans (28). It codes for glycoproteins that are crucial in discriminating self from non-self and hence plays critical role in activating innate and adaptive immune system (29). Unlike other genes, the variants in MHC code for proteins which differ by about 20 amino acids (30) and this genetic diversity is a major contributor for difference in immune responses among different individuals. The best example for this variability is the slow progression of HIV to AIDS depending upon the presence of specific *HLA-B* and *HLA-C* alleles (31). This suggests that certain environmental pathogens may remain asymptomatic but might have evoked immune responses in the individual without any phenotypic manifestation. Similarly we presume that specific variants in MHC might evoke a response that might play a direct or indirect role in triggering predisposition risk of NSHL. The role of MHC on predisposition to NSHL can be understood by investigating its expression in ear. It has been demonstrated that the inner ear expresses MHCII in response to increased IFNγ levels following infection (32). Class II MHCs are concerned with presentation of peptides to Helper T cells. This clearly indicates that MHCII expression is crucial in protection of inner ear against invading pathogens and environmental stressors, and MHCII variants may have a critical role in varying the levels of predisposition to NSHL.

Heat shock proteins (HSPs) are molecular chaperons whose activity is enhanced in response to cellular stress induced chiefly by pathogens (33). These molecular chaperons aids in assembly of MHC peptide complex. Thus, HSP70 helps in antigen presentation and processing (34). Similarly HSPs induce the production of proinflammatory cytokines and it has also been shown that the levels of HSPs increase significantly in sudden sensorineural hearing loss (SSNHL) (35). HSP70 is located adjacent to HLA class III on 6p21.3 region (35). Previous studies have found out a strong Linkage disequilibrium (LD) between class I *HLA B7, B38, B44*, class II *HLA-DR2, DR4*, and 90kb variant of *HSP70* (36). *HSP1A* variant rs1043618 has been found to be associated with noise induced hearing loss, which can be extended to variants in strong LD with *HLA* (37). This suggests a strong role of HLA and HSPs to play a role in etiology of NSHL via its variants or its altered expression in inner ear.

Pattern recognition receptors (PRRs) are crucial components of antigen presentation like MHCs. PRRs are integral parts of innate immunity. PRRs recognize conserved patterns called PAMPs in non-self-organisms like flagellar proteins, cell wall components of bacteria, and fungi as well as viral nucleic acids in addition to DAMPs associated with cellular stress, inflammation, and so on. PRRs are subdivided into four classes as TLRs, NLRs, CLRs, and RLRs of which TLRs and CLRs are transmembrane proteins whereas NLRs and RLRs are cytosolic proteins (38). Besides recognizing molecular patterns they are also inducers of inflammatory cytokines. Polymorphisms in TLRs have been already found to be associated with middle ear infections like otitis media (39). It has been demonstrated that CMV infection is associated with *TLR2* variants (40). *TLR2, TLR4*, and *TLR9* are also known to have risk SNPs for survivors of meningitis patients (41). In case of Meniere's disease the allelic variants of *TLR10* were found to influence the causation of disease. The infections and its immunological after effects are perceived by the PRRs present on the surface of inner ear especially through TLRs and so we suspect a strong role for the variants in these PRRs to play a role in predisposition of disease. The varying expression of PRRs can cause difference in susceptibility to NSHL as well as affect the expression of its numerous downstream target proteins thus playing a role in causation.

## Cytokine Imbalance and Predisposition Risk

Cytokines are key mediators of inflammatory process and are driven by induction of PRRs. PRRs on activation can induce the expression of MAPK, AP1 as well as NFkB. NFkB is normally present in inactive form in cytoplasm by the inhibitory protein IkB. IkB on activation by various stimuli phosphorylates NFkB and gets degraded in the proteasome (42). The then active NFkB moves into the nucleus and activates the pro inflammatory cytokine cascade (22, 43). Cytokines are crucial in permeability of endothelium, bone resorption, osteoclast formation as well as inflammation. Inflammation is an essential process to evade and protect the host from pathogens or tissue damage by vasodilation and recruitment of immune cells at the target site. Inflammation initially considered as a protective mechanism is now negatively linked to metabolic, developmental, neurodevelopmental, and neurodegenerative disorders. Increased amount of proinflammatory cytokines has been reported in brain tissues of patients' with neuropsychological pathologies such as Alzheimer's, Stroke, Multiple sclerosis, and psychiatric disorders (44). At the genetic level also, variants in pro inflammatory cytokines has been found to be associated with Schizophrenia, Aneurysm, and Alzheimer's (45–47). Pro inflammatory cytokines are also inducers of reactive oxygen species thus rendering oxidative stress along with inflammation.

Difference in susceptibility toward diseases is attributed fully or in parts to cytokine variants (48). This can be clearly visualized in the difference in prevalence of infectious diseases in different parts of the world as has reported that the genotype frequencies of cytokine variants significantly varied in different populations (49). *TNF*α variant rs1800630 and *TNFRSF1B* variant rs1061624 were found to be associated with age related hearing loss in elderly population of Japanese origin (50). There are only mice model studies in relation to elevated levels of proinflammatory cytokines especially IL1β and IL18, in relation to age related hearing loss. Cochlear inflammation and oxidative stress are found to be the main reasons for noise induced hearing loss (51). In the case of SSNHL also elevated levels of TNF α, IL2, IL8, and IL6 has been reported (52), which are also likely to be induced by CMV infection. Two recent studies in very small samples have shown evidences of mutation in *IFNLR1* to be associated with Autosomal Dominant NSHL (53) and *CCL2* variants rs31900 and rs1024611 to be risk variants in etiology of CMV associated sensorineural hearing loss (54). These facts indicate that the balance of cytokines is crucial in cochlea and cochlea is highly vulnerable to inflammation. We hypothesize that the balance of cytokines is very crucial during the first trimester of pregnancy, an increased pro inflammatory state can damage the cochlear structure, while decreased anti-inflammatory state makes it vulnerable to pathogens and any of these can increase the pre-disposition risk of NSHL. The evidences also suggest that genetics variants in cytokine genes are key to define the threshold of inflammatory response.

**Figure 1:**
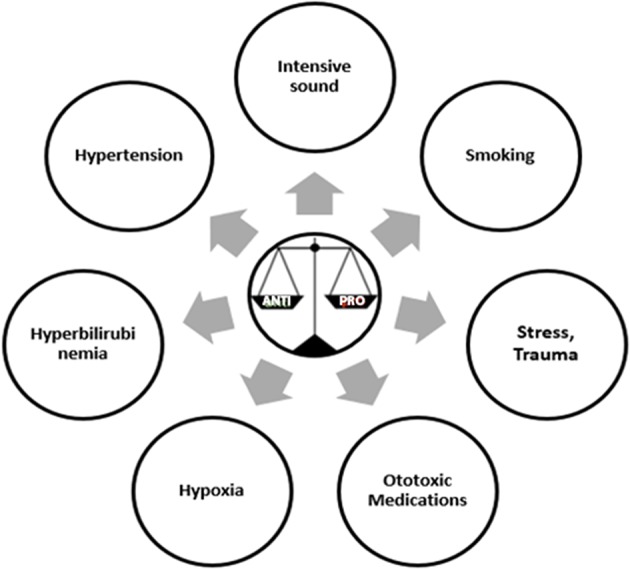
Sterile inflammatory factors that can induce a risk for Non-Syndromic Hearing Loss.

Pathogens are known to be the primary trigger for the initiation of inflammatory process. Other than microbial factors there are several other factors that can also induce an inflammatory response and these are termed as sterile inflammatory factors (55). Some of these sterile inflammatory factors as shown in (Figure 1) are also reported to induce hearing loss (56, 57). The relationship between hypertension and cytokines is like a chicken and egg story. Hypertension is associated with higher levels of TNF α, IL6, IL1β, IL17, and C Reactive Protein (CRP) (58). Stress, smoking and noise are also known to stimulate inflammation by modulating the three key cytokine players of inflammation TNFα, IL1β, and IL6 (59–61). While hypoxia is shown to elevate the levels of cytokines, chemokines, and adhesion molecules (62). There are several medications known to have ototoxic effects but the mechanism by which each drug causes ototoxicity is not well-established. In case of cisplatin, a tumor drug, known to have ototoxic effect elevates the production of proinflammatory cytokines via STAT6 pathway (63). Bilirubin another important regulator of immune responses of the host has been demonstrated to increase the production of anti-inflammatory cytokine IL10 and reduce the expression of proinflammatory cytokine TNFα (64). Aging process itself is associated with subtle increase in inflammation, with an elevated levels of pro inflammatory cytokines and diminished levels of anti-inflammatory cytokines called “Inflammaging” (65). Interestingly, a recent article has shown hypertension, ototoxicity, hypoxia, birth trauma, and severe jaundice to be risk factors for the development of congenital hearing loss (57). Even the medications such as glucocorticoids, immunosuppressants, and biologics are used as therapeutics for sensorineural hearing loss and autoimmune inner ear diseases (56). All these drugs have anti-inflammatory and immune suppressive functions. These evidences further strengthened our hypothesis that the different environmental risk factors may be playing a role in the etiology of NSHL via the immune responses. We also suggest that other environmental factors should also be considered other than the routine viral factors while investigating the etiology of NSHL. Genetic variants in these immune response genes and their regulation by environmental factors might be key to understanding and addressing the differential impact on pathology of hearing loss.

## Interactome of Immune Response and Deafness Genes in Nshl

**Figure 2:**
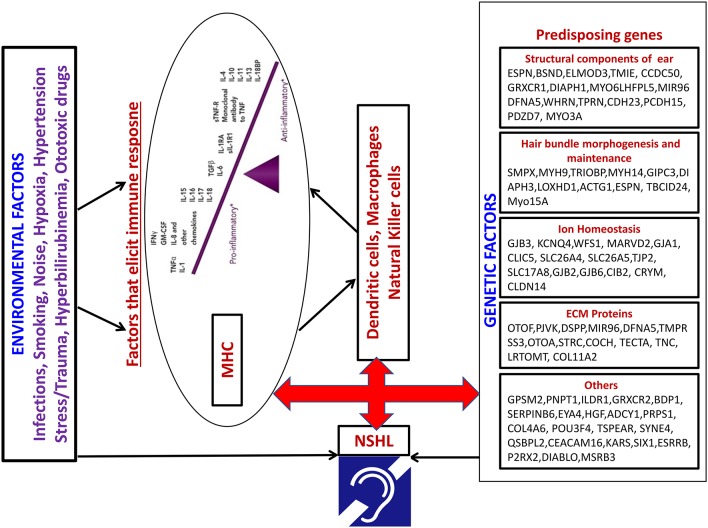
Model depicting the possible interaction between Environmental factors and pre-disposing NSHL genes through immune mediated pathway.

The interaction between candidate genes of NSHL and immune response genes has not yet been investigated. Recently few transcription factors have been identified that can play a critical role in NSHL through immune response modulation. *EYA4* is a novel transcription factor found to be associated with autosomal dominant hearing loss in exome sequencing studies (66). *EYA4* have been shown to be critical in Interferon mediated immune responses especially Type I *IFN* genes. Similarly, *ESRRb* has been recently reported to be associated with hearing loss (67), which is capable of inducing self-renewal and pluripotency in Embryonic stem cells devoid of cytokines (68). This indicates a compensatory role of *ESRRb* in the absence of cytokines. It would be interesting to identify how immune response alterations can impact the modulation of pathway that is critical to hearing loss. The possible mechanism of interaction between the two through immune mediated pathway resulting in hearing loss is demonstrated in Figure 2. The role of the expression of candidate genes in the inner ear in presence of an immune reaction is a heavily unexplored area. Understanding the activation of immune response regulation and its cascading effects on the gene expression that is crucial in causation of disease pre-disposition would be the need of the hour.

## Concluding Remarks

The complexity of the NSHL makes it difficult to unravel the complete etiology of the disease. Therefore, there is a need to identify a unifying mechanism that might be central to the presently pursued investigations. In this regard, based on the evidences presented, it seems perturbations in immune response could be critical in resolving the mystery behind the etiology of NSHL. Congenital hearing loss occurs during the first trimester of pregnancy and any alterations in immune profile at this stage can be crucial for normal development of inner ear structures. We propose a strong role of immune response genes that may either directly impact inner ear structures during development or indirectly by altering the expression profile of genes that are crucial for inner ear development, resulting in NSHL pathology.

## Author Contributions

MB conceptualized the topic. MB and KS wrote the article.

### Conflict of Interest

The authors declare that the research was conducted in the absence of any commercial or financial relationships that could be construed as a potential conflict of interest.

## Supplementary Material

Supplementary Figure 1 Loci associated with hearing loss. Colour codes indicate the functional role of the loci.

## Abbreviations

AP1: activated protein 1
CLR: C type lectin receptor
CMV: cytomegalo virus
DAMP: damage associated molecular pattern
HSP: heat shock protein
LD: linkage disequilibrium
MAPK: mitogen activated protein kinase
MHC: major histocompatibilty complex
NFKB: nuclear factor kappa B
NLR: nucleotide binding oligomeisation domain like receptors
NSHL: Non-Syndromic Hearing Loss
SSNHL: sudden sensorineural hearing loss (SSNHL)
PAMP: pathogen associated molecular patterns
PRR: pattern recognition receptors
RLR: rig 1 like receptor
TLR: Toll like receptor
ESRRb: estrogen related receptor b.
