# Supplementary figures and images for: An Immunological Perspective to Non-syndromic Sensorineural Hearing Loss
Source: Front Immunol. 2019 Dec 11;10:2848. doi: 10.3389/fimmu.2019.02848 (PMC6919260; doi:10.3389/fimmu.2019.02848)

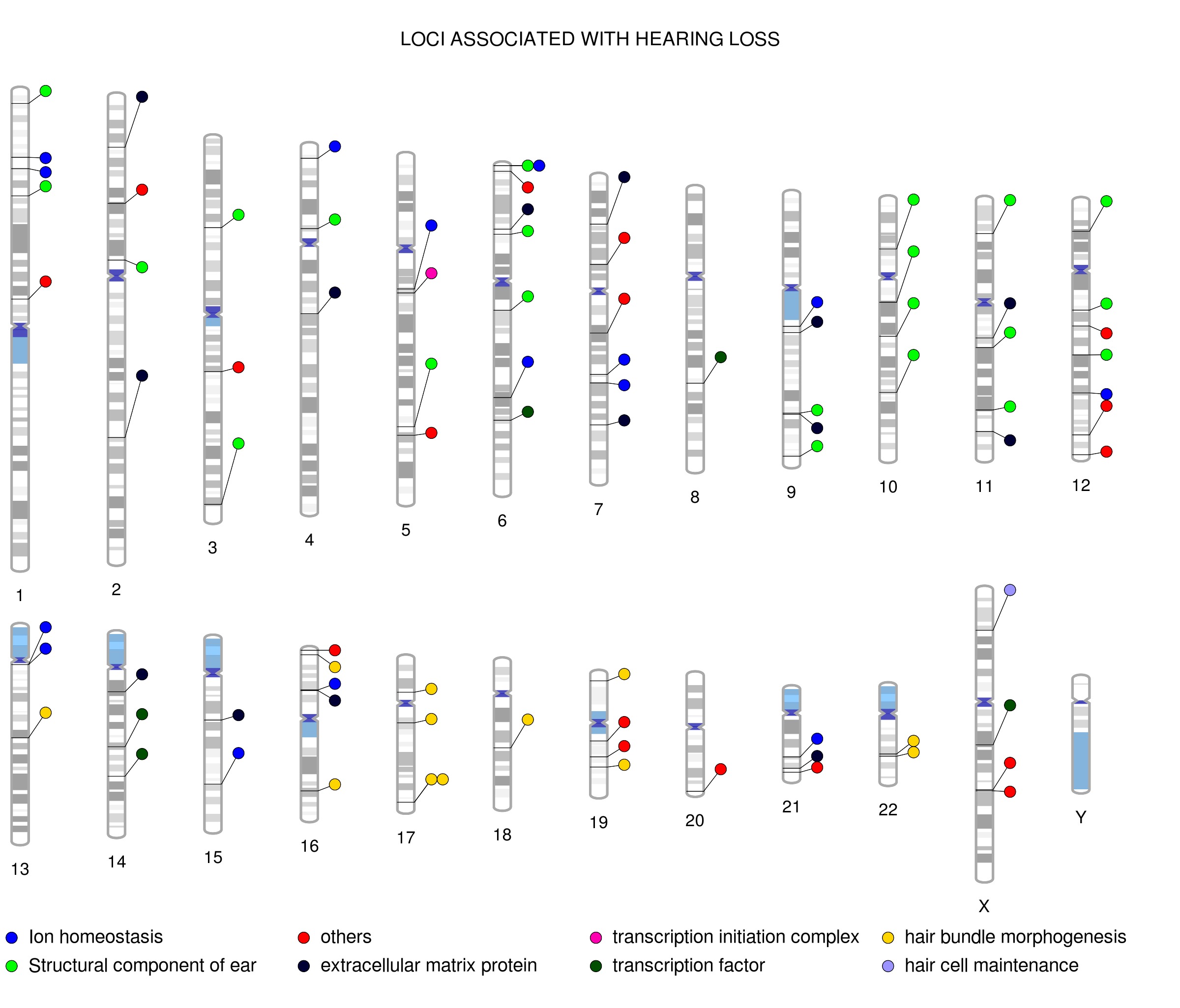

Supplement: Supplementary Figure 1 — Loci associated with hearing loss. Colour codes indicate the functional role of the loci. [file Image_1.jpg]
